# Etiology, characteristics, and outcomes of community-onset necrotizing fasciitis in Korea: A multicenter study

**DOI:** 10.1371/journal.pone.0218668

**Published:** 2019-06-20

**Authors:** Tark Kim, Seong Yeon Park, Yee Gyung Kwak, Jiwon Jung, Min-Chul Kim, Seong-Ho Choi, Shi Nae Yu, Hyo-Lim Hong, Yong Kyun Kim, Se Yoon Park, Eun Hee Song, Ki-Ho Park, Oh Hyun Cho, Sang-Ho Choi, the Korean SSTI Study Group

**Affiliations:** 1 Department of Internal Medicine, Soonchunhyang University Bucheon Hospital, Bucheon, Republic of Korea; 2 Department of Internal Medicine, Dongguk University Ilsan Hospital, Goyang, Republic of Korea; 3 Department of Internal Medicine, Inje University Ilsan Paik Hospital, Goyang, Republic of Korea; 4 Department of Internal Medicine, Ulsan University Hospital, University of Ulsan College of Medicine, Ulsan, Republic of Korea; 5 Department of Internal Medicine, Chung-Ang University Hospital, Seoul, Republic of Korea; 6 Department of Internal Medicine, Soonchunhyang University Cheonan Hospital, Cheonan, Republic of Korea; 7 Department of Internal Medicine, Daegu Catholic University Medical Center, Daegu, Republic of Korea; 8 Department of Internal Medicine, Inje University Haeundae Paik Hospital, Pusan, Republic of Korea; 9 Department of Internal Medicine, SoonChunHyang University Seoul Hospital, Seoul, Republic of Korea; 10 Department of Internal Medicine, GangNeung Asan Hospital, GangNeung, Republic of Korea; 11 Department of Internal Medicine, Kyung Hee University Hospital, Kyung Hee University School of Medicine, Seoul, Republic of Korea; 12 Department of Internal Medicine, GyeongSang National University Changwon Hospital, Changwon, Republic of Korea; 13 Department of Infectious Diseases, Asan Medical Center, University of Ulsan College of Medicine, Seoul, Republic of Korea; George Washington University, UNITED STATES

## Abstract

**Background:**

Necrotizing fasciitis (NF) is a serious skin and soft tissue infection causing high mortality. Investigating region specific epidemiologic factors associated with NF is important for establishing appropriate treatment strategies. This multicenter study was done to provide an update of the microbial etiology, clinical characteristics, and outcomes of NF in Korea.

**Materials and methods:**

A retrospective cohort of adult patients with NF was established using patient data from 13 general hospitals between January 2012 and December 2015 in Korea. We evaluated microbial etiology and clinical characteristics to identify risk factors associated with in-hospital mortality; analyses were performed using binary logistic regression models.

**Results:**

A total of 161 patients with NF were included. The most common underlying disease was diabetes mellitus (66 cases, 41.0%). A total of 148 organisms were isolated from 119 (73.9%) patients. Enteric Gram-negative organisms (36 patients) were the most common pathogen, followed by *Staphylococcus aureus* (30 patients) and streptococci (28 patients). Methicillin-resistant *Staphylococcus aureus* (MRSA) was identified in 6.2% (10/161) of patients. Of 37 enteric Gram-negative isolates tested, 26 (70.3%) isolates were susceptible to ceftriaxone. The in-hospital mortality rate was 22.4%. Intensive care unit admission, septic shock, and Gram-negative organism infections were significantly associated with in-hospital mortality, and surgery was not a favorable prognostic factor.

**Conclusions:**

As initial empirical antibiotics, glycopeptides against MRSA and broad-spectrum antibiotics against third-generation cephalosporin-resistant organisms should be considered for patients with community-onset NF in Korea.

## Introduction

Necrotizing fasciitis (NF) is a severe soft tissue infection that is characterized by fulminant tissue destruction and systemic toxicity. Even with the advancement of medical technology, NF still results in high mortality. In recent studies, 20–30% of mortality has been reported in patients with NF [1–5]. Underlying conditions such as old age and liver cirrhosis have been known as important non-modifiable factors associated with mortality in patients with NF [3, 6]. Urgent surgical intervention and appropriate antibiotics usage are modifiable factors for favorable prognosis in NF [5, 7]. Early surgical intervention is especially important for preventing limb loss due to ischemia and hypoxia caused by NF [8]. Also, it is important to know which patients are at risk to NF and which organisms are major etiologic pathogens for NF in order to establish effective treatment strategies.

Several microbiological epidemiology studies of NF have been conducted, identifying various microorganisms as etiologic pathogens of NF. In the United States, methicillin-resistant *Staphylococcus aureus* (MRSA) has been reported as one of the important pathogens in NF [9, 10], and anti-MRSA antibiotics such as glycopeptide are recommended as an empirical regimen in NF [11]. However, because microbial epidemiology can differ by region and vary over time, other treatment guidelines may be more adequate. Also, data on antimicrobial susceptibility are necessary to determine strategies for empirical antibiotics for NF. Therefore, we conducted a multicenter study to determine the etiology, clinical characteristics, outcomes of community-onset NF in Korea.

## Materials and methods

### 1. Study design & population

A retrospective cohort study was conducted among thirteen teaching hospitals in the Republic of Korea. Initially, we identified patients who were diagnosed with the Korean Standard Classification Disease and Cause of Death code for NF between January 2012 and December 2015. Episodes of community-onset NF, which were defined as infections that developed before or within the first 48 hours of hospital admission, were only included. After comprehensive review of clinical, radiologic, and intraoperative findings, we included cases that were clinically compatible with NF. Patients younger than 18 years old were excluded from the analysis.

### 2. Data collection

Using a standardized protocol, patient demographics (age and sex), underlying diseases (diabetes mellitus, liver cirrhosis, end-stage renal disease, alcoholism, solid tumor, hematologic malignancy, and immunocompromised state, and site of infection were searched. End-stage renal disease was defined as when renal dialysis was needed for more than 3 months. Cases were categorized into healthcare-associated infections if any one of the following conditions were satisfied: (1) previous admission within 3 months for 2 days or more before episode; (2) previous intravenous antibiotics use, chemotherapy, or nursing care at home within 1 month before presenting with NF; (3) the previous hemodialysis within 1 month before episode; or (4) if the patient’s residence was a nursing facility [12]. All other patients were categorized as a community-acquired infection. Intensive care unit (ICU) admission during hospitalization and septic shock were used as the severity indices. Septic shock was defined in accordance with the most recent international consensus (Sepsis-III) [13]. Also, laboratory findings at the time of admission such as white blood cell count, platelet count, level of creatinine, and of C-reactive protein were collected. The results of cultures using blood, pus, and intra-surgical specimens were also reviewed. Surgical intervention as a treatment modality and in-hospital mortality as an outcome indicator was also investigated.

### 3. Microbiologic methods

Initially, sheep blood, chocolate, and MacConkey agar plates were used for isolation of aerobic organisms. In the case of closed pus specimens, anaerobic culture was routinely carried out. Brucella agar plates on which pus was inoculated were incubated in an anaerobic chamber or commercial pouch. Tissue specimens were inoculated into enriched thioglycolate broth for the isolation of anaerobes. Microorganism identification was performed using standard methods at each hospital in which the quality control of microbiological tests has passed the evaluation of accredited institutions. Susceptibility testing was done using the microdilution method, and results were interpreted according to the National Committee for Clinical Laboratory Standards guidelines [14].

### 4. Statistical analysis

All statistical analyses were calculated using SPSS Statistics version 14.0 (SPSS, Chicago, IL, USA). The following analysis was used depending on the nature of variables: Chi-square test or Fisher’s exact test for categorical variables, the Mann-Whitney U test for continuous variables. The covariates with a significance of *P* < 0.1 were used included in the binary logistic regression models for multivariate analyses. Variables known to be risk factors for mortality in NF were also included in the multivariate analysis. Using a receiver operating characteristics curve, continuous variables were converted to categorical variables. All tests were two-tailed, and differences were considered significant at *P* < 0.05.

## Results

### 1. Study population & clinical characteristics

A total of 161 patients with NF were included during the study period. A median of 10 patients (interquartile range [IQR] 5–18 patients) was enrolled from each hospital. Radiologic studies were done in 123 (76.4%) patients: 68 (42.2%) patients underwent computed tomography, 67 (41.6%) patients underwent magnetic resonance imaging, and 6 (3.7%) patients underwent ultrasonography. Demographics, underlying disease/condition, and clinical characteristics of enrolled patients are summarized in Table 1. The median age was 59 years (range, 48–71 years) and 108 patients were male (67.1%). The most common underlying disease was diabetes mellitus (66 patients, 41.0%), followed by alcoholism (19 patients, 11.8%) and liver cirrhosis (18, 11.2%). Twenty three (14.3%) patients were categorized as having a healthcare-associated infection. NF was most frequently identified in lower extremities (103 patients, 64.0%).

**Table 1:** Demographics, underlying disease/conditions, and clinical characteristics of patients with community-onset necrotizing fasciitis.

Variable	No. of patients (%) (N = 161)
Demographics	
Median age, year (IQR)	59 (48–71)
Sex, male	108 (67.1)
Underlying diseases or conditions	
Diabetes mellitus	66 (41.0)
Alcoholism	19 (11.8)
Liver cirrhosis	18 (11.2)
Solid tumor	13 (8.1)
End-stage renal disease	5 (3.1)
Hematologic malignancy	2 (1.2)
Immunocompromised^a^	11 (6.8)
Healthcare-associated infection	23 (14.3)
Previous admission within 3 months for more than 2 days before the episode	16 (9.9)
Previous IV antibiotics, chemotherapy or nursing care at home within 1 month before the episode	11 (6.8)
Previous hemodialysis within 1 month before the episode	5 (3.1)
Nursing facility	6 (3.7)
Site of infection	
Lower extremity	103 (64.0)
Upper extremity	19 (11.8)
Other	39 (24.2)
Severity	
ICU admission during hospitalization	69 (42.9)
Septic shock	53 (32.9)
Laboratory findings at the time of admission, median (IQR)	
White blood cell count, /mm^3^	13,840 (9,600–20,100)
Platelet count, 10^3^ /mm^3^	208,000 (110,000–320,000)
Creatinine level, mg/dL	1.1 (0.8–1.8)
C-reactive protein, mg/dL	15 (6.7–25.9)
Surgery	116 (72.0)
In-hospital mortality	36 (22.4)

^a^Patients were defined as immunocompromised if they had human immunodeficiency virus or acquired immunodeficiency syndrome; or if they had received solid organ or hematopoietic stem cell transplantation, chemotherapy within 6 weeks, systemic steroids equivalent to or higher than 20 mg of prednisone for 2 weeks, or other immunosuppressive agents within 2 weeks before hospitalization.

Abbreviations: IQR, interquartile range; IV, intravenous; ICU, intensive care unit

### 2. Microbial etiology

Microbial etiologies of NF are shown in Table 2. A total of 119 patients (73.9%) had one or more identifiable pathogens. Microbiologic tests were performed in 154 (95.7%) patients: 138 (85.7%) for blood culture, 97 (60.2%) for intraoperative specimens and 45 (28.0%) for aspiration or biopsy. The positive rates of culture according to specimen source were as follows: 77.3% (75/97) from intraoperative specimens, 68.9% (31/45) from aspiration or biopsy specimens and 37.0% (51/138) from blood cultures. A total of 148 organisms were isolated, and two or more pathogens were isolated in 19 (11.8%) patients. Organisms identified in polymicrobial infections are listed in Table 3. Among 19 patients with polymicrobial infections, 8 patients had both aerobic Gram-positive cocci and Gram-negative organism infections.

**Table 2:** Identified microbial etiologies in patients with community-onset necrotizing fasciitis.

Variables	Number of Patients (%) (N = 161)^a^
Any pathogen	119 (73.9)
Aerobic Gram-positive organisms	69 (42.6)
*Staphylococcus aureus*	30 (18.6)
Methicillin-susceptible *S*. *aureus*	20 (12.4)
Methicillin-resistant *S*. *aureus*	10 (6.2)
Streptococci	28 (17.4)
Viridans streptococci	12 (7.5)
*Streoptococcus agalactiae/dysgalactiae*	10 (6.2)
*Streptococcus pyogenes*	9 (5.6)
Coagulase-negative staphylococci^b^	8 (5.0)
Enterococci	7 (4.3)
Aerobic Gram-negative organisms	55 (34.2)
Enteric gram-negative rod	36 (22.4)
*Escherichia coli*	19 (11.8)
*Klebsiella* species.	12 (7.5)
*Proteus* species.	4 (2.5)
*Enterobacter* species.	2 (1.2)
*Citrobacter* species.	1 (0.6)
Non-enteric gram-negative rod	20 (12.4)
*Vibrio vulnificus*	10 (6.2)
*Pseudomonas* species.	5 (3.1)
*Acinetobacter* species.	4 (2.5)
*Aeromonas* species.	2 (1.2)
Anaerobe and *Candida* species^c^	10 (6.2)

^a^Denominator of percentage is the total number of enrolled patients. A total of 148 organisms were isolated from 119 (73.9%) patients, and polymicrobial infections were found in 19 of 119 (16.0%) patients. Microbiologic tests were performed in 154 (95.7%) patients: 138 for blood culture, 97 for intraoperative specimens and 45 for aspiration or biopsy. The positive rates of culture according to specimen source were as follows: 77.3% (75/97) from intraoperative specimens, 68.9% (31/45) from aspiration or biopsy specimens and 37.0% (51/138) from blood cultures.

^b^Half (4/8) of coagulase-negative staphylococci infection were polymicrobial infections

^c^Three isolates of *Bacteroides* species, one isolate of *Clostridium* species, two isolates of peptostreptococci, two isolates of *Prevotella* species, one isolate of *Bacillus* species, one isolate of *Fusobacterium* species, one isolate of *Kocuria* species, and two isolates of *Candida* species.

**Table 3:** Isolates from patients with community-onset necrotizing fasciitis by polymicrobial infection.

Study No.	Gram-positive cocci	Gram-negative rod	Others
1	*Enterococcus faecalis*, *Streptococcus*. *anginosus*		
2	*Streptococcus mitis/oralis*		*Clostridium* species
3	Coagulase-negative staphylococci	*Citrobacter freundii*	
4	Methicillin-susceptible *Staphylococcus*. *aureus*	*Escherichia coli*	
5	Methicillin-suscpetible *S*. *aureus*, viridans streptococci		
6		*Klebsiella pneumoniae*	*Bacteroides fragilis*
7	Methicillin-susceptible *S*. *aureus*	*E*. *coli*	
8		*K*. *pneumoniae*, *E*. *coli*	*Bacteroides uniforminis*, *Peptostreptococcus* species, *Prevotella oralis*
9	Coagulase-negative staphylococci, *E*. *faecium*		*Candida glabrata*
10	Coagulase-negative staphylococci, *Enterococcus*	*E*. *coli*, *Pseudomonas aeruginosa*	
11	Coagulase-negative staphylococci	*Acinetobacter baumannii*	
12	*E*. *faecalis*		*Fusobacterium* species
13		*K*. *pneumoniae*, *A*. *baumannii*, *E*. *coli*	
14	*E*. *faecalis*	*E*. *coli*	
15	*S*. *mitis/oralis*, viridans streptocci		
16	*S*. *agalactiae*	*E*. *coli*	
17	Methicillin-susceptible *S*. *aureus*, *S*. *agalactiae*, *S*. *anginosus*		
18	Methicillin-resistant *S*. *aureus*		*Candida albicans*, *Peptostreptococcus* species, *P*. *oralis*
19	Methicillin-susceptible *S*. *aureus*	*Klebsiella oxytoca*	

Aerobic Gram-positive organisms were identified in 42.6% (69/161) of patients. Of them, *S*. *aureus*, found in 30 (18.6%) patients, were the most common pathogens, followed by streptococci in 28 (17.4%) patients and enterococci in 7 (4.3%) patients. Among 30 isolates of *S*. *aureus*, MRSA was identified from 6.2% (10/161) patients (community-acquired cases, 6.5% [9/138] vs. healthcare-associated cases, 4.3% [1/23]; *P* > 0.99).

Enteric Gram-negative organisms were identified in 22.4% (36/161) of patients. Antimicrobial susceptibility of enteric Gram-negative rods isolated from patients with community-onset necrotizing fasciitis is shown in Table 4. Of these isolates, 70.3% (26/37) were susceptible to ceftriaxone, 73.0% (27/37) to cefepime, 91.9% (34/37) to piperacillin/tazobactam, 100% (37/37) to ertapenem, 70.3% (26/37) to ciprofloxacin, 97.1% (33/34) to amikacin, and 64.0% (16/25) to trimethoprim/sulfamethoxazole. The proportion of enteric Gram-negative isolates susceptible to ceftriaxone was not significantly different between patients with healthcare-associated infections (13.4%, 3/23 patients) when compared to community-acquired cases (16.7%, 23/138 patients; *P* > 0.99). Of 21 non-enteric Gram-negative organisms isolated from 12.4% (20/161) patients, *Vibrio vulnificus* was the most commonly identified organism. Of 10 patients with *V*. *vulnificus* infection, 9 (90%) patients were diagnosed with chronic alcoholism and 7 (70%) patients had liver cirrhosis. All of *V*. *vulnificus* isolates were susceptible to ceftriaxone. Of 4 *Pseudomonas* species and 3 *Acinetobacter* species for which antimicrobial susceptibility information were available, 57.1% (4/7) of organisms were susceptible to ceftazidime and piperacillin/tazobactam,

**Table 4:** Antimicrobial susceptibility of enteric Gram-negative rods isolated from patients with community-onset necrotizing fasciitis.

Enteric G(-) rod	HCA (%)	Ceftriaxone (%)	Cefepime (%)	Piperacillin /tazobactam (%)	Ertapenem (%)	Ciprofloxacin (%)	Amikacin (%)	TMP /SMX (%)
^a^*Escherichia coli* (n = 19)	33.3	61.1	61.1	88.9	100	55.6	100	53.3
*Klebsiella* spp.(n = 12)	16.7	91.7	91.7	100	100	100)	100	72.7
*Proteus* spp (n = 4).	50.0	50.0	50.0	75.0	100	25.0	75.0	NA
*Enterobacter* spp.(n = 2)	0	50	100	100	100	100	100	NA
*Citrobacter* spp.(n = 1)	0	100	100	100	100	100	100	100
Total (n = 38)	26.3	70.3	73	91.9	100	70.3	97.1	64.0

^a^Antibiogram of one *Escherichia*. *coli* isolate was not availabe

Abbreviations: HCA, healthcare-associated; G(-), Gram-negative; TMP/SMX, trimethoprim/sulfamethoxazole; spp., species.

### 3. Outcomes

Overall in-hospital mortality was 22.4% (36/161). Median duration of hospitalization until death was 8 days (IQR 2–25). Surgery was not performed in 45 (28.0%) patients with NF. Reasons for not performing surgery included: improvement in condition after antibiotic therapy, despite delayed diagnosis, in 17 patients; high risk of morbidity and mortality in 9 patients; refusal of surgery in 6 patients; mortality before decision in 6 patients; and unknown reasons in 7 patients. Surgical intervention was not a significant risk factor for in-hospital mortality (receiving surgery 20.7% [24/116] vs. not receiving surgery 26.7% [12/45]; *P* = 0.41).

Mortality according to each pathogen were as follows: 22.2% (2/9) for *S*. *pyogenes*, 20% (2/10) for *S*. *agalactiae/dysgalactiae*, 16.7% (2/12) for viridans streptococci, 14.3% (1/7) for enterococci, 25.0% (2/8) for coagulase-negative staphylococci, 13.3% (4/30) for *S*. *aureus*, 31.6% (6/19) for *E*. *coli*, 58.3% (7/12) for *Klebsiella* species, 70.0% (7/10) for *V*. *vulnificus*, 0% (0/5) for *Pseudomonas* species, and 75.0% (3/4) for *Acinetobacter* species. Patients with Gram-negative organism infections, when compared with patients with Gram-positive organism infections, were more likely to have chronic alcoholism (21.8% [12/55] vs. 6.6% [7 /106]; *P* = 0.008), liver cirrhosis (21.8% [12/55] vs. 5.7% [6/106]; *P* = 0.003), ICU admission during hospitalization (61.8% [34/55] vs. 33.0% [35/106]; *P* = 0.001), and septic shock (50.9% [28/55] vs. 23.6% [25/106]; *P* = 0.001). Mortality of patients with enteric Gram-negative infections did not significantly differ according to the third-generation cephalosporin resistance (34.6% [9/26] for the third-generation cephalosporin susceptible pathogen vs. 45.4% [5/11] for non-susceptible, *P* = 0.74). Polymicrobial infection was also not associated with increased mortality (26.0% [26/100] for monomicrobial infection vs. 31.6% [6/19] for polymicrobial infection; *P* = 0.59).

Healthcare-associated infection, alcoholism, liver cirrhosis, end-stage renal disease, septic shock, ICU admission during hospitalization, Gram-negative organism infections, white blood cell count ≤ 8,000/mm^3^, platelet count ≤ 85,000/mm^3^, and creatinine ≥ 1.2 mg/dL were associated with in-hospital mortality in the univariate analysis (Table 5). In multivariate analysis, ICU admission during hospitalization (adjusted odds ratio [aOR], 30.38; 95% confidence interval [95% CI], 3.50–263.67), septic shock (aOR, 8.46; 95% CI, 2.31–30.93), and Gram-negative organism infections (aOR, 3.20; 95% CI, 1.08–9.48) were independent risk factors for in-hospital mortality.

**Table 5:** Risk factors associated with an in-hospital mortality of patients with community-onset necrotizing fasciitis.

Variables	No. of death/No. of episodes (%)	*P*	Adjusted Odds Ratio (95% Confidence Interval)	*P*
	Total (N = 161)			
Demographics				
Age ≥ 60 year	22/77 (28.6)	0.09		
Sex, male	28/108 (25.9)	0.16		
Underlying diseases or conditions				
Alcoholism	8/19 (42.1)	0.04		
Liver cirrhosis	9/18 (50.0)	0.006		
End-stage renal disease	4/5 (80.0)	0.009		
Healthcare-associated infection	9/23 (39.1)	0.06		
Site of infection				
Lower extremity	25/103 (24.3)	0.56		
Severity				
Septic shock	31/53 (58.5)	< 0.001	8.46 (2.31–30.93)	0.001
ICU admission during hospitalization	35/69 (50.7)	< 0.001	30.38 (3.50–263.67)	0.002
Laboratory findings at the time of admission				
White blood cell count ≤ 8000/mm^3^	17/28 (60.7)	< 0.001		
Platelet count ≤ 85000/mm^3^	19/31 (61.3)	< 0.001		
Creatinine ≥ 1.2 mg/dL	29/70 (41.4)	< 0.001		
Surgery	24/116 (20.7)	0.41		
Etiology				
Gram-negative organism infection	23/55 (41.8)	< 0.001	3.20 (1.08–9.48)	0.04
Polymicrobial infection	6/19 (31.6)	0.38		

Abbreviation: ICU, intensive care unit

## Discussion

Our findings show that community-onset NF resulted in high in-hospital mortality among the thirteen Korean hospitals participating in the study and that severity was independently associated with mortality. We also identified that enteric Gram-negative organisms were the most common etiology, followed by *S*. *aureus*. About 70% of enteric Gram-negative organism infections were susceptible to third-generation cephalosporin, while about 30% of *S*. *aureus* infections were MRSA. This study is the largest multicenter investigation of current microbial etiology and clinical characteristics of community-onset NF in Korea. It is expected that this study can be used to establish new treatment strategies for community-onset NF in Korea.

For many years, community-acquired MRSA has been of interest as an emerging pathogen in many countries. In a study conducted at six academic hospitals in Texas, United States, MRSA was the most frequently identified organism in mono-microbial necrotizing soft tissue infection (35%) although there was a significant variation between hospitals (4–41%) [10]. About 30% of *S*. *aureus* causing NF was methicillin-resistant in a hospital in Iowa, United States [9]. In a study conducted in 12 European countries, MRSA was identified as the causative microorganisms in 14.6% (169/1190) in complicated skin and soft tissue infection [15]. European Antimicrobial Resistance Surveillance Network by 28 participating countries reported that MRSA accounted for 16.7% of all *S*. *aureus* isolates in complicated skin and soft tissue infection in 2012 [16]. Methicillin resistance of *S*. *aureus* isolates was even more than 50% in Romania and Portugal [16]. These epidemiologic studies suggest that MRSA is an important pathogen causing NF both in the United States and European countries. Although the proportion of MRSA-associated NF cases does not appear to be as high in Korea (6.2%) as in the United States and Europe, considering the high risk of mortality of NF, it cannot be neglected.

MRSA rates were higher among healthcare-associated infections, compared to community-acquired infections [17]. This is also true in Korea [18, 19]. Surprisingly, risk factors associated with MRSA in NF have not been evaluated well. Our study did not find that healthcare-associated infections were associated with MRSA infections in NF. This is consistent with the findings that the invasion of community-acquired MRSA strains such as USA-300 into healthcare facilities has blurred the traditional concept of healthcare-associated infections [20]. The lack of a significant association of healthcare-associated infection with MRSA infection suggests that NF by MRSA can occur unpredictably regardless of the setting. Therefore, our study supports the guidelines recently published by the Korean Society of Infectious Diseases, which recommend glycopeptides as an initial empirical regimen for NF [21].

Choosing appropriate initial empirical antibiotics is critical when treating NF. In a recent study done in Spain, 25.3% of patients received inappropriate initial empirical antibiotics and inadequate empirical antibiotics usage was associated with in-hospital mortality (OR, 6.04; 95% CI, 5.40–370.73) [7]. Antibiotic susceptibility data obtained in our study is useful data for selecting appropriate initial empirical antibiotics. Especially since data on antibiotics susceptibility of Gram-negative organisms causing NF has been limited. In our study, *Pseudomonas* species and *Acinetobacter* species, which are notorious for their ability to develop antimicrobial resistance, were isolated in 9 (5.6%) patients with NF (Table 2). In addition, enteric Gram-negative organisms not susceptible to ceftriaxone were isolated in 11 (6.8%) patients with NF (Table 4). Therefore, Gram-negative organisms resistant to third-generation cephalosporins should be considered as potential etiologies of NF in Korea.

It is difficult to determine at which level empirical antibiotics against MRSA and 3rd cephalosporin antibiotics resistant Gram-negative organisms should be used in NF. Narrow spectrum antibiotics increase the risk of inappropriate antibiotics, while broad-spectrum antibiotics increase the risk of antimicrobial resistance. Cost-benefit studies are required to precisely define this level, but it seems to be impossible to conduct such studies in NF. Considering that NF is a severe disease that causes high mortality, we believe that the presence of MRSA in more than 5% of NF cases may be sufficient to warrant the use of empirical antibiotic therapy. In addition, the healthcare-associated infection might not be a useful criterion to choose empirical antibiotics in NF. Therefore, our findings support the guideline recently published by the Korean Society of Infectious Diseases which recommends a glycopeptide plus piperacillin-tazobactam or carbapenems as initial empirical regimens for treating NF [21].

Although surgical intervention was not associated with lower in-hospital mortality, the importance of early surgical intervention should not be denied. The effectiveness of antibiotics can be limited in NF because the delivery of antibiotics can be compromised by tissue ischemia and hypoxia [8]. Indeed, owing to survival bias, it is difficult to establish the effectiveness of surgery on improving survival in a retrospective study. Therefore, the results of this study that surgery did not affect in-hospital mortality should be interpreted with caution. We believe that early surgery should be considered in all patients with NF.

Septic shock was an important risk factor for in-hospital mortality. Early appropriate antibiotics and surgical intervention are very important for patients with septic shock. Although we identified patients who did not receive surgical intervention survived, our findings should not be interpreted as suggesting that urgent therapeutic interventions can be delayed in patients without severe manifestations of NF. Early diagnosis and treatment with broad-spectrum empirical antibiotics and surgical intervention should still be done in patients with NF, regardless of septic shock status.

In our study, Gram-negative organism infection was an independent risk factor for in-hospital mortality. The severity of Gram-negative organism infection should be interpreted in the context of host and microbiologic factors. For example, patients with Gram-negative organism infections were more likely to have alcoholism or liver cirrhosis. Among Gram-negative organisms, *V*. *vulnificus* and *K*. *pneumoniae* have been well known as virulent pathogens causing high mortality [22, 23].

This study has some limitations. First, some isolates may be a result of wound colonization rather than pathogenic infection. Second, because the main purpose of this study was to investigate recent etiologies of NF in Korea, data on therapeutic interventions were not fully investigated. The use of appropriate initial empirical antibiotics and their role in affecting in-hospital mortality could not be analyzed. Therefore, our findings with respect to risk factors of in-hospital mortality should be interpreted cautiously.

In conclusion, glycopeptides against MRSA and broad-spectrum antibiotics against third-generation cephalosporin-resistant organisms should be considered as initial empirical antibiotics for patients with community-onset NF in Korea.

## Compliance with ethical standards

All procedures performed in studies involving human participants were in accordance with the ethical standards of the institutional and/or national research committee and with the 1964 Helsinki declaration and its later amendments or comparable ethical standards.

## Ethical approval

This work was approved by the Institutional Review Board (IRB) of Soohchunhyang University Buchoen Hospital (2017-01-001), Dongguk University Ilsan Hospital (2017-01-012), Inje University Ilsan Paik Hospital (ISPAIK 2017-01-010), Chung-Ang University Hospital (1704-002-16061), Soonchunhyang University Cheonan Hospital (2017-02-021), Daegu Catholic University Medical Center (CR-17-019), Inje University Haeundae Paik Hospital (2017-01-029), SoonChunHyang University Seoul Hospital (2017-03-012), GangNeung Asan Hospital (2017-02-002), GyeongSang National University Changwon Hospital (2019-04-003), Asan Medical Center (S2019-0854-0001). Kyunghee University and Ulsan University conducted research based on the IRB approval of Soonchunhyang University Bucheon Hospital. Informed consent was waived since this work was a retrospective study without intervention and did not involve extra clinical specimens. This study was conducted using the format recommended by the Strengthening the Reporting of Observational Studies in Epidemiology (STROBE) guidelines.

## Supporting information

S1 Data (SAV)

S2 Data (SAV)
